# Congenital Zika Syndrome and Disabilities of Feeding and Breastfeeding in Early Childhood: A Systematic Review

**DOI:** 10.3390/v15030601

**Published:** 2023-02-22

**Authors:** Evangelia Antoniou, Paraskevi Eva Andronikidi, Panagiotis Eskitzis, Maria Iliadou, Ermioni Palaska, Maria Tzitiridou-Chatzopoulou, Nikolaos Rigas, Eirini Orovou

**Affiliations:** 1 Department of Midwifery, University of West Attica, Agioy Spyridonos 28, 12243 Egaleo, Greece; 2 Faculty of Medicine, University of Crete, 71003 Crete, Greece; 3 Department of Midwifery, University of Western Macedonia, 50200 Ptolemaida, Greece

**Keywords:** Zika virus, congenital Zika syndrome, feeding difficulties, breastfeeding difficulties

## Abstract

Background: The Zika virus outbreak has affected pregnant women and their infants. Affected infants develop microcephaly and other congenital malformations referred to as congenital Zika syndrome. The neurological manifestations of congenital Zika syndrome may result in some feeding disorders, including dysphagia, swallowing dysfunction and choking while feeding. The aim of this study was to assess the prevalence of feeding and breastfeeding difficulties in children with congenital Zika syndrome and to estimate the risk of developing feeding disabilities. Methods: We searched PubMed, Google Scholar and Scopus for studies published from 2017 to 2021. From the total of 360 papers, reviews, systematic reviews, meta-analyses and publications in languages other than English were excluded. Therefore, the final sample of our study consisted of 11 articles about the feeding/breastfeeding difficulties of infants and children with congenital Zika syndrome. Results: Infants and children with congenital Zika syndrome were likely to suffer from feeding difficulties at various levels, including breastfeeding. Dysphagia problems ranged from 17.9% to 70%, and nutritional and non-nutritive suckling of infants was also affected. Conclusions: In addition to continuing to investigate the neurodevelopment of affected children, future research should also focus on the severity of factors influencing the degree of dysphagia, as well as the impact of breastfeeding on the child’s overall development.

## 1. Introduction

In early 2015, some incidents of patients with symptoms of rash, mild fever, arthralgia and conjunctivitis were reported in northeastern Brazil. After the Chikungunya and dengue infections were ruled out, Zika virus (ZIKV) was detected by reverse transcription-polymerase chain reaction from the sera of eight patients [1]. After Brazil, ZIKV spread rapidly to the Americas, and by March 2017, more than 80 countries worldwide were reporting Zika infection [2]. Zika virus is a flavivirus transmitted by the bite of Aedes aegypti and Aedes albopictus mosquitoes in humans [3]. By the end of September 2015, an increasing number of infants with microcephaly were noted in the northeast of Brazil, thus leading researchers to link vertical transmission in pregnancy with severe fetal malformations [4,5]. These congenital malformations are referred to as congenital Zika syndrome (CZS) and may occur after symptomatic or asymptomatic infection in the mother [6], mainly during the first trimester of pregnancy [7]. CZS includes severe microcephaly, in which the skull has partially collapsed; there is decreased brain tissue, including subcortical calcifications; damage to the back of the eye (including focal retinal pigmentary mottling and macular scarring); and hypertonia soon after birth and clubfoot or arthrogryposis [8,9,10,11]. CZS has also been associated with other central nervous system (CNS) abnormalities, such as brain atrophy and asymmetry, absent or abnormally formed brain structures, neuronal migration disorders (effects on neocortical layer formation such as brain calcifications, lissencephaly, ventriculomegaly and cerebellar hypoplasia) and hydrocephalus. Microcephaly is a malformation in which the size of the head is smaller than expected for the age and gender (more than two standard deviations (SDs) below the mean). It is divided into two types: (a) primary microcephaly, which develops before 32 weeks of gestation or birth, and (b) secondary microcephaly, which develops after gestation or birth [12]. Primary microcephaly, the most important focus of Zika virus, is generally caused due to disturbed neurogenesis (mitosis or progenitor cell function or death of neural progenitors). ZIKV infection can lead to both primary and secondary microcephaly (postnatal onset). However, the secondary form of microcephaly usually relates to the postnatal development and maturation of neurons (reduction in dendrites and synaptic connections, or defects in myelination, or even both mechanisms acting in concert) [12,13]. The sooner ZIKV exposure occurs during pregnancy, the sooner the fetus’s head stops developing and the head circumference meets the clinical definition for congenital microcephaly. More specifically, infection during the first or second trimester increases the risk of congenital microcephaly [14,15] owing to increased placental permissiveness at these periods [16] However, there are cases of children exposed to ZIKV in utero who were born normocephalic and developed a progressive neurodevelopmental delay due to retardation of brain development [17,18].

Swallowing is a complex behavior, involving both reflex and volitional activities implicating more than 30 nerves and muscles [19]. There are four stages that describe the movement of the bolus during swallowing: (a) preparatory stage—after taking the food from the mouth, the bolus is placed on the back of the surface of the tongue, and then the oral cavity is sealed by the contact of the soft palate and the tongue in order to prevent the bolus from entering the pharynx before swallowing; (b) oral propulsive stage—there are differences in the consumption of liquid and solid food. After liquid consumption, the posterior buccal cavity is sealed by tongue–palate contact, while solid food is held in the buccal cavity with the tongue and soft palate moving circularly with jaw movement, allowing buccal–pharyngeal communication [20,21]; (c) pharyngeal stage—this stage occurs rapidly and is characterized by the entry of food into the pharynx and esophagus with simultaneous protection of the airways larynx–trachea, during the passage of food; and (d) esophageal stage—at this stage, relaxation is observed during swallowing to allow the bolus to pass into the stomach. The upper part of the esophagus (cervical) consists of striated muscles, and its lower part (thoracic) of smooth muscles. Therefore, bolus transport in the lower esophagus is regulated by the autonomic nervous system [21]. In addition, eating, swallowing and breathing are connected and synergistic in normal people. More specifically, breathing stops slightly during swallowing due to the closure of the airway and the elevation of the soft palate and due to the nervous suppression of breathing by the brainstem [22]. There are a wide variety of diseases that can be responsible for dysphagia, which are distinguished by structural damage to the area, psychiatric disorders, iatrogenic causes or neurological disorders (neurogenic dysphagia) [23]. Neurogenic dysphagia is mainly caused by damage to the basal ganglia of the brain and cerebral cortex, cerebellum, stem and lower cranial nerves [24].

According to the above, the neurological manifestations of CZS in infants may result in some feeding disorders (dysphagia, choking while feeding), which complicate the already existing situation by increasing the risk of morbidity and mortality through malnutrition [25,26]. For this reason, the WHO guidance recommends that infants born to mothers with suspected, probable or confirmed antenatal ZIKV infection, with or without microcephaly, should be evaluated for neurological abnormalities and feeding problems at follow-up monitoring at least at 3, 9 and 24 months of age [27]. It was observed that even CZS infants with neurologic manifestations that were not very severe (without microcephaly) exhibited dysphagia at birth [28]. A study published in 2019 [29] showed that over 50% of mothers of children with CZS reported breastfeeding difficulties, and this explained the high prevalence of early weaning of ZIKV children. More specifically, less than 20% of children were breastfed continuously at 12 months, while 35% of children without microcephaly breastfed continuously at the same point. Therefore, it seems that in children with microcephaly changes in swallowing, oral motor coordination and suckling make breastfeeding difficult.

Breastfeeding has many short-term and long-term benefits for both mother and infant. Exclusive breastfeeding for the first 6 months is associated with a significant reduction in infection and diseases [30], as well as cognitive, language and motor development in infants [31]. Currently, there are no data reports of ZIKV transmitted to neonates through breastfeeding [32], and for this reason, the WHO, in order to prevent ZIKV transmission, published a recommendation for infants born to mothers with suspected, probable or confirmed ZIKV infection that is similar to that for other infants. This recommendation advocates breastfeeding within the first hour of birth, exclusive breastfeeding for 6 months, introduction of complementary foods and continuation of breastfeeding until the age of 2 years [33]. However, this guideline did not take into account the special feeding requirements of infants affected by CZS.

Thus, the need for a systematic review of the literature was noted in order to find relevant articles concerning the feeding (including breastfeeding) of children with the special needs of CZS. The aim of this study was to assess the prevalence of feeding and breastfeeding in children with CZS and to estimate the risk of developing feeding disabilities.

Nevertheless, feeding abilities differ between neonates, infants and children. For example, suckling and swallowing are already observed from the fetal life stage (14–15th week). Coordination of suckling, breathing and swallowing is developed in neonates from birth. In addition, they adjust suckling according to the different types of milk flow (breast or bottle milk). At the age of 2 months, the infants can move the food from the spoon to the back of the mouth, and between 4 and 6 months, when the infant learns to control the tongue, purees or smooth foods are introduced into the infant’s diet. At the period of 6–12 months the front teeth emerge, so they can chew soft pieces, while between 12 and 24 months, they can cope with most food textures and most foods in the family meal [34].

## 2. Materials and Methods

### 2.1. Inclusion and Exclusion Criteria

We included prospective studies as well as cohort and cross-sectional studies from 2017 onwards that evaluated the association between CZS and feeding/breastfeeding disabilities. This review followed the guidance of the Preferred Reporting Items for Systematic Reviews and Meta-Analyses (PRISMA) [35]. We excluded all studies that (a) were review articles (systematic or not) and letters to the editor; (b) enrolled infants who were not infected by ΖIKV; (c) enrolled infants whose neurodevelopment malformations were not related to CZS, (d) did not report data documenting the strength of the association between CZS and feeding and (e) were not written in the English language.

### 2.2. Exposure/Outcomes

We defined exposure as all infants diagnosed with CZS. Furthermore, all infants had some degree of neurological impairment, with developmental delays at all levels, hypertonia and pyramidal and extrapyramidal signs.

To investigate our outcome, we included all studies that provided information on feeding and breastfeeding in infants and children with CZS.

### 2.3. Search Strategy

We searched all published English articles on the following databases: Scopus, PubMed/Medline and Google Scholar, and conducted a literature review from 3 August to 26 October 2022. The terms we used were: infants with CZS OR ZIKV infected infants OR ZIKV and congenital malformations AND breastfeeding outcomes OR breastfeeding problems; infants with CZS OR ZIKV infected infants OR ZIKV and congenital malformations AND feeding problems OR feeding disabilities; and infants with CZS OR ZIKV infected infants OR ZIKV and congenital malformations AND nutritional status OR nutritional deficiency.

### 2.4. Study Selection

Two authors (E.A. and E.O.) evaluated the titles and abstracts independently. Then, the full texts of all shortlisted articles were retrieved and evaluated for eligibility using the predefined inclusion and exclusion criteria. There were no disagreements; therefore, they were not reviewed by another author.

### 2.5. Methοdological Quality and Risk of Bias

No significant difference appeared in the methodological quality and risk of bias of the articles. The main problems in all studies were that exposure was not assessed more than once over time, and the outcome assessors were not blinded to the exposure status of the participants because the types of studies did not support this design (Table 1). The quality assessment tool we used was developed by NHLBI and Research Triangle Institute International in 2013 in Washington, USA. It consists of a set of customized quality assessment tools designed to help reviewers focus on concepts that are central to the internal validity of a study. The tools were based on quality assessment methods, concepts and other tools developed by researchers at the Agency for Healthcare Research and Quality (AHRQ) Centers for Evidence-Based Practice, the Cochrane Collaboration, the USPSTF, the Scottish Intercollegiate Guidelines Network Lines and the National Center for Reviews and Dissemination of Health Services [36].

Furthermore, selection bias in all articles was low because participants did not differ in their basic characteristics. Performance bias did not exist because no group of children was exposed to factors that affected the results. The way results were collected was reliable across all articles so detection bias was not present. Finally, no data were hidden from any article so the results were not distorted (reposting bias).

## 3. Results

**Figure 1:**
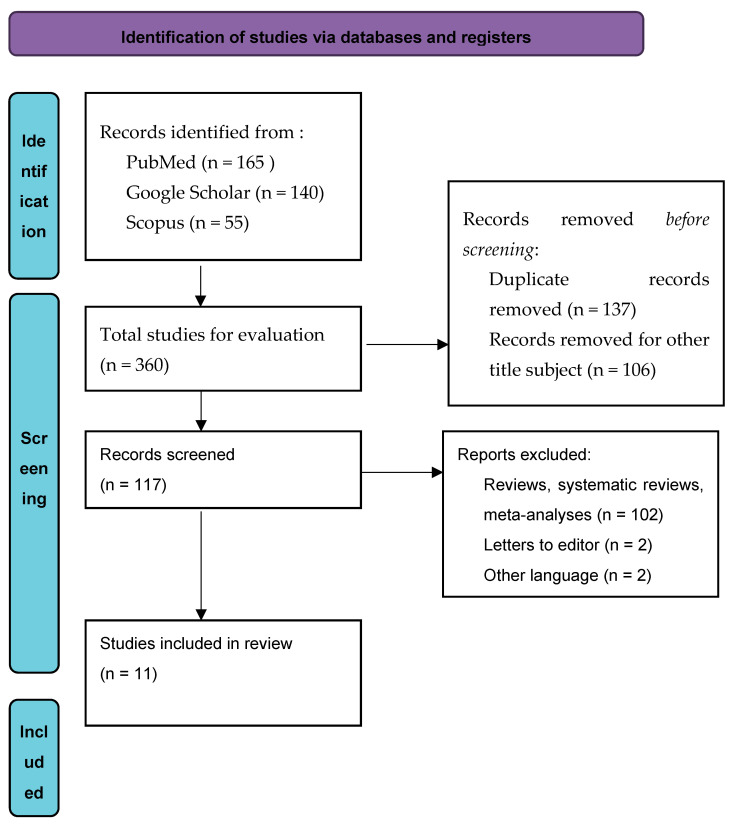
Flow diagram of included papers.

The initial search of the databases found 360 papers. After removing all duplicate and “other title subject” papers, 117 remained to be evaluated. Subsequently, 106 papers were removed as they were reviews, systematic reviews, meta-analyses, letters to the editor or published in another language than English. Finally, 11 articles were included in the systematic review (Figure 1).

Four studies had a cohort design [4,29,37,43], three cross-sectional [38,40,42] and two longitudinal [39,41] and two studies were case series [18,25] (Table 2). All research articles were conducted in Brazil, where the highest prevalence of ZIKV occurred [44]. All articles provided data from a serological analysis of anti-Zika antibodies in mothers and infants. All children and infants had undergone imaging tests to document neurological disorders. We also extracted information on mothers’ sociodemographics and, where possible, information on pregnancy data regarding ZIKV exposure. The children’s age ranged between 1 and 24 months.

We identified feeding disabilities in all articles. One of them [37] associated ZIKV with dysphagia, while the remaining 10 articles [4,18,25,29,38,39,40,41,42,43] referred to dysphagia with breastfeeding problems. Finally, all the participants had, in addition to eating disorders, severe neurological disabilities associated with the ZIKV syndrome [4,18,25,29,37,38,39,40,41,42,43].

### 3.1. Feeding Difficulties

Among the remaining articles, the results of the first one [37] showed that feeding difficulties, severe motor disability, hearing and vision abnormalities and seizures tended to coexist. Dysphagia problems ranged from 17.9% to 70% [4,38] in children with CZS. More specifically, 17.9% of 56 infants infected with ZIKV manifested hypersalivation, choking and reflux in the Soares study [4], while none of the control group of infants showed similar symptoms. Furthermore, in Silva’s study [37], 47% of children with CZS presented feeding difficulties since their diet consisted of liquid, pureed or strained foods. The onset of dysphagia began after the third month of life in eight of the nine infants with CZS in another study [25], while swallowing was abnormal in the entire sample. However, seven of nine infants were unable to swallow a sufficient volume of contrast to allow esophageal transit time analysis. Another important finding was a significant relationship between height at 12 months of age in infants with CZS and dysphagia [40], while there were difficulty swallowing (60%), excessive salivation (57.8%), a requirement for highly processed food, enteral nutrition through gastrostomy and lower than normal weight in a significant number of children with CZS in the study by Oliveira [42]. Finally, Ferreira’s study [38] showed that 70% of children with CZS manifested eating difficulties.

### 3.2. Breasteeding Difficulties

The infant’s ability to suckle is a necessary condition for the initiation of feeding through breastfeeding. However, nutritional and non-nutritive suckling problems in children with CZS result in breastfeeding difficulties. Abnormal swallowing was confirmed in some studies [25,41,43], while during the 6th month of the infants’ life, breastfeeding rates decreased considerably [4,18,25,29,41,42], and only the minority of them were still breastfeeding at 12 months [29,40,41]. In some studies, an above average percentage of infants did not breastfeed [29,38] and, as a result, a high percentage of infants used a bottle [4,41]. In addition, in the case of control studies, it appeared that lower percentages of infants with microcephaly breastfed exclusively compared to the controls [4,42,43] or had good weight gain [39].

However, a worse nutritional status can be counteracted by the beneficial properties of breastfeeding. The weight of infants who were fed with their mother’s milk, either through breastfeeding, expressing breast milk or using donor milk, was much better than the infants who drank formula milk [39,40].

## 4. Discussion

The results of this study showed that children with CZS were likely to suffer from feeding difficulties at various levels, including breastfeeding. Several studies have shown that children with CZS have feeding difficulties, which are related to the insufficient suckling ability and dysfunction in the orofacial muscles [45,46]. Feeding difficulties are a well-known phenomenon in children with neurological disabilities. Insufficient control and coordination of the muscles reduce the effectiveness of suckling, chewing and swallowing [47]. Dysphagia in children with neurological disorders is characterized by significant variability, which is determined by the degree of neurological damage [48]. Severe CZS is associated with severe cerebral palsy involving the cortical and subcortical regions, basal ganglia and brainstem. These conditions can cause disturbances in any phase of swallowing [25].

Swallowing that follows the breastfeeding reflex is a voluntary process of the infant that requires optimal functioning of the cerebral cortex, which is not fulfilled in children with CZS [49]. For this reason, a high percentage of infants, according to our results, did not initiate breastfeeding after birth, and as a result, they used the bottle. The feeding bottle is compressed during feeding for slow and continuous swallowing, where milk drips into the infant’s oral cavity without requiring any special effort by the infant [41].

According to our results, the majority of infants who breastfed did not continue to do so past 6 months, and few breastfed for the whole first year of their life. However, we could not determine whether the infants with CZS had dysphagia problems or whether the early introduction of formula feeding was a cause of breastfeeding cessation. Additional explanations could be the mother’s lack of cooperation, her mental distress and the lack of breastfeeding promotion programs for mothers of children with CZS [50,51]. In addition, the use of pacifiers is a widespread cultural habit when raising Brazilian children [52]. Moreover, offering the pacifier to children with CZS may be a means of calming and comforting these infants, whose irritability and crying has been linked to their condition [25]. At the same time, however, the use of baby bottles and pacifiers has been associated with early weaning [53].

There is no recommendation to stop breastfeeding in mothers exposed to ZIKV or in infants exposed intrauterine to ZIKV [54,55], but there can be significant difficulties in establishing and maintaining breastfeeding. Nevertheless, our results confirm the beneficial effects of breastfeeding through maintaining a normal body weight. Breast milk has been shown to have a positive impact on weight gain and weight retention compared to formula milk [56]. In addition, there is convincing evidence for the beneficial properties of breast milk given that the infant’s brain is sensitive to nutrition [57].

A key limitation of the study was that it was conducted only in Brazil; in the future, more cohort studies on the effects of CZS on children’s nutrition from all affected countries are needed. In addition, the registration protocol was not peer reviewed in PROSPERO.

## 5. Conclusions

For the majority of children with CZS, feeding is a difficult process. In addition to continuing to investigate the neurodevelopment of affected children, future research should also focus on the severity of factors influencing the degree of dysphagia, as well as the impact of breastfeeding on the child’s overall development. It is considered imperative to develop education and psychological support programs for families who have children with CZS. In addition, breastfeeding should be supported by any method possible (breastfeeding, expressing breast milk or donor milk).

## Figures and Tables

**Table 1:** Methodological quality and risk of bias of included articles.

	Studies										
Criteria	Leal [25], 2017 Brazil	Satterfield -Nash [37], 2017 Brazil	Ferreira [38], 2018 Brazil	dos Santos [29], 2019 Brazil	dos Santos [39], 2019 Brazil	Soares [4], 2019 Brazil	Carvalho- Sauer [40], 2020 Brazil	Cavalcanti [41], 2020 Brazil	Peçanha [18], 2020 Brazil	Oliveira [42], 2020 Brazil	Medeiros [43], 2021 Brazil
1. Research question clearly stated	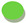	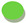	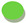	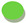	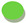	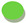	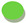	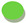	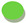	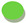	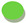
2. Study population clearly specified and defined	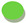	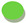	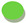	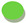	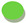	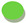	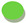	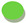	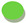	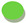	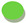
3. Participation rate of eligible persons at least 50%	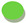	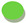	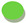	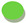	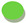	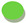	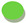	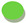	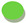	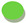	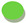
4. Same or similar study populations, prespecified inclusion/exclusion criteria	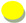	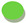	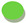	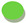	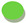	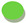	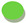	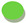	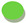	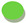	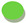
5. Sample size justification	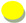	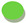	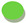	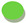	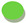	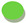	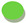	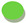	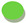	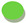	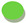
6. Exposure of interest measured prior to the outcome	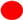	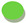	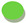	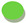	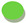	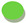	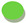	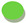	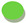	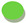	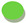
7. Sufficient timeframe between exposure and outcome	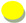	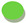	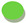	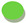	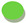	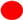	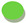	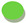	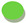	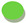	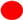
8.The study examined different levels of exposure as related to the outcome	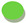	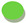	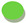	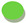	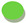	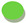	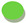	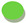	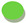	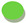	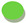
9. Clearly defined exposure measures	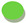	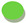	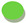	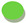	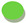	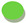	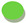	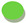	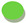	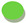	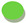
10. Exposure assessed more than once over time	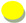	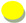	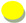	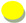	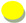	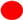	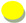	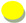	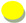	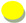	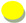
11. Outcome measures clearly defined	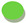	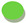	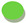	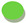	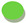	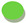	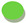	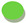	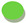	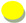	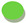
12. Outcome assessors blinded to the exposure status	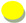	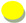	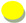	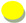	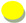	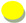	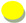	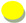	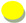	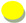	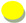
13. Loss to follow-up after baseline 20% or less	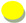	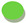	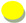	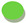	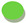	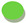	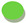	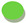	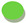	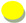	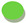
14. Confounding variables measured and adjusted statistically	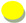	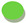	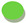	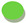	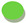	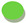	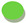	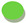	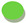	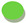	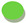

Notes: 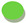: low risk of bias; 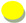: some concerns (unclear); 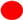: high risk of bias.

**Table 2:** Research studies included in the review.

Author/Year/ Country	Design	N	Exposure	Data Collection	Feeding Outcomes
Leal [25], 2017 Brazil	A descriptive, retrospective case-series study	9	Children with dysphagia and CZS 8–24 months of age	From the medical records of three tertiary care institutions	Feeding dysphagia and problems with breastfeeding were manifested after the third month of life in eight of the nine infants. Abnormal swallowing in all infants.
Satterfield-Nash [37], 2017 Brazil	Cohort study	19	Children with CZS 19–24 months of age	ZODIAC research	47% of children presented feeding difficulties.
Ferreira [38], 2018 Brazil	A descriptive cross-sectional study	34	Children 21 months of age with microcephaly due to CZS	From rehabilitation services	More than 70% of children with microcephaly had severe difficulty eating, 52% of them were not breastfeed
dos Santos [29], 2019 Brazil	Data from a cohort study “Fernandes Figueira National Institute of Women, Children and Adolescent Health—Oswaldo Cruz Foundation”	65	Infants 12–23 months of age with microcephaly	A public institute	80% of the infants were not exclusively breastfed until the 6th month. 53.6% of the mothers reported difficulties with breastfeeding. At the age of 12–23 months, few infants continued breastfeeding.
dos Santos [39], 2019 Brazil	A longitudinal descriptive study	21	Full-term neonates exposed to ZIKV intrauterine	A public neonatal intensive care unit	CZS was associated with worse nutritional status. Mean weight of infants consuming only human milk (via breastfeeding and/or expressing breast milk and pasteurized milk from the milk bank) tended to be higher than that of infants consuming only infant formula.
Soares [4], 2019 Brazil	Cohort study	115	56 infants who were exposed intrauterine to ZIKV and 59 who were unexposed, all 1–3 months of age	A part of a large cohort study based in Rio de Janeiro, Brazil	17.9% of infected infants presented dysphagia (hypersalivation, choking and reflux) compared to the non-infected infants. By the third month of age, 48.3% of exposed infants receiving formula milk compared to 22.2% of unexposed infants.
Carvalho-Sauer [40], 2020 Brazil	Cross-sectional study	46	Children up to 12 months of age with CZS	Sourced from 22 municipalities in the State of Bahia by convenience sampling	56.8% of children had dysphagia. There was a positive correlation between breastfeeding time and weight at 3 and 6 months of age, and only a minority of these children were still breastfeeding at 12 months.
Cavalcanti [41], 2020 Brazil	Observational, longitudinal study	98	Children 2–17 months of age with CZS	Interviews with mothers of children with CZS from two rehabilitation centers	89.9% of children were breastfed at birth; by the age of 6 months, 36.6% continued breastfeeding, 48% had swallowing difficulty and 27.8% had suckling difficulties; use of bottle was reported for 89.9%.
Peçanha [18], 2020 Brazil	An exploratory case series	84	Asymptomatic children exposed to ZIKV intrauterine (range 6–18 months)	Outpatient clinic at Instituto Fernandes Figueira (IFF)-Fundação Oswaldo Cruz (Fiocruz)	Exclusive breastfeeding was maintained in 58.3% of children up to 6 months.
Oliveira [42], 2020 Brazil	Cross-sectional study	45	45 children with CZS and 50 healthy controls, all 6 months of age	Three rehabilitation centers	Difficulty swallowing (60%), excessive salivation (57.8%) and non-exclusive breastfeeding until 6 months (84.4%). Ultraprocessed food intake, lower weight and enteral nutrition through gastrostomy or jejunostomy were noted in children with CZS.
Medeiros [43], 2021 Brazil	Retrospective cohort with nested case-control study	86	Two groups of neonates, with microcephaly (n = 43) and without microcephaly (n = 43) (from birth to the 37th day of life)	Data were collected from a maternity hospital in northeastern Brazil	During hospitalization, 34.9% of neonates with microcephaly breastfed exclusively, in contrast to the control group, which breastfed at a rate of 47.4%. A nasogastric feeding tube was used in 23.3% of the microcephaly group, while in the control group, it was used in 7.9%. 58% of the neonates in the control and 70% of the neonates in the control group (without microcephaly) were taken to the maternal breast as soon as they were born.

## Data Availability

Not applicable.
